# Diversity of acid stress resistant variants of *Listeria monocytogenes* and the potential role of ribosomal protein S21 encoded by *rpsU*

**DOI:** 10.3389/fmicb.2015.00422

**Published:** 2015-05-08

**Authors:** Karin I. Metselaar, Heidy M. W. den Besten, Jos Boekhorst, Sacha A. F. T. van Hijum, Marcel H. Zwietering, Tjakko Abee

**Affiliations:** ^1^Top Institute Food and NutritionWageningen, Netherlands; ^2^Laboratory of Food Microbiology, Wageningen UniversityWageningen, Netherlands; ^3^NIZO Food ResearchEde, Netherlands; ^4^Bacterial Genomics Group, CMBI Centre for Molecular and Biomolecular Informatics, Radboud University Medical CenterNijmegen, Netherlands

**Keywords:** heterogeneity, glutamate decarboxylase, phenotypic characterization, whole genome sequencing, trade-off, SNP analysis

## Abstract

The dynamic response of microorganisms to environmental conditions depends on the behavior of individual cells within the population. Adverse environments can select for stable stress resistant subpopulations. In this study, we aimed to get more insight in the diversity within *Listeria monocytogenes* LO28 populations, and the genetic basis for the increased resistance of stable resistant fractions isolated after acid exposure. Phenotypic cluster analysis of 23 variants resulted in three clusters and four individual variants and revealed multiple-stress resistance, with both unique and overlapping features related to stress resistance, growth, motility, biofilm formation, and virulence indicators. A higher glutamate decarboxylase activity correlated with increased acid resistance. Whole genome sequencing revealed mutations in *rpsU*, encoding ribosomal protein S21 in the largest phenotypic cluster, while mutations in *ctsR*, which were previously shown to be responsible for increased resistance of heat and high hydrostatic pressure resistant variants, were not found in the acid resistant variants. This underlined that large population diversity exists within one *L. monocytogenes* strain and that different adverse conditions drive selection for different variants. The finding that acid stress selects for *rpsU* variants provides potential insights in the mechanisms underlying population diversity of *L. monocytogenes*.

## Introduction

*Listeria monocytogenes* is a ubiquitous microorganism, which may encounter a variety of environmental conditions during its transmission from soil to the human gastro-intestinal tract (Freitag et al., 2009). In the last decades, many researchers have demonstrated that microbial populations are not isogenic but comprise phenotypic and genotypic heterogeneity. The presence of subpopulations, each of which is prepared to survive and multiply under a specific condition allows the organism to survive in a wide range of environmental conditions. Van Boeijen et al. (2010) showed that a set of high hydrostatic pressure (HHP) resistant variants of *L. monocytogenes* LO28 had different phenotypic and genotypic characteristics. The variants showed highly increased resistance toward multiple types of stress, different degrees of motility and different growth rates. Despite the fact that the presence of stable resistant subpopulations has been clearly demonstrated not only for *L. monocytogenes* (Karatzas and Bennik, 2002; Van Boeijen et al., 2008) but also for other microorganisms (Hauben et al., 1997; Sagarzazu et al., 2013), the mechanisms behind the increased stress resistance of these subpopulations is poorly understood. A mutation in the class III heat shock repressor *ctsR* was shown to be responsible for the increased HHP resistant phenotype for some *L. monocytogenes* variants (Karatzas et al., 2003; Van Boeijen et al., 2010; Van Boeijen et al., 2011). Mutations in *ctsR* can lead to a defect in the repression of a number of chaperone encoding genes like *clpC* which results in transcription of these stress response genes. *ctsR* variants have been isolated after HHP stress in several *L. monocytogenes* strains and is the only mutation identified in stress resistant variants until now. Interestingly, the HHP resistant variants also showed increased resistance to other types of stress, including heat, and acid stress. This led to the hypothesis that also heat and acid stress could select for this type of variants. Both heat and acid resistant variants have been isolated recently (Van Boeijen et al., 2011; Metselaar et al., 2013) and for most of these heat resistant variants a mutation in *ctsR* was found which appears to be responsible for its increased heat resistance. However, the acid resistant variants are not further characterized yet and it is not known if these variants harbor the same mutation or if acid stress selects for a different type of variants. One of the most important and well-studied systems in acid resistance of *L. monocytogenes* is the glutamate decarboxylase (GAD) system (Cotter et al., 2001). An extracellular glutamate molecule is imported by an antiporter in exchange for an intracellular γ-aminobutyrate (GABA) molecule. Each molecule of glutamate is decarboxylated by a decarboxylase to produce a molecule of GABA. During this process a proton is consumed. This results in an increase of the cytoplasmic pH and thus protects the cell against the acidic environmental conditions (Feehily and Karatzas, 2013). Recently, a new model for the GAD system has been proposed by Karatzas et al. (2012), in which two different GAD systems were discriminated. The intracellular GAD system uses metabolically synthesized glutamate and the extracellular system uses environmental glutamate which is accumulated in the cell by dedicated transporters. The intracellular system for the production of GABA seems to be important for the LO28 strain in Brain Heart Infusion (BHI), which we also used in our study. Our study aims to get more insight in the population diversity of *L. monocytogenes* LO28 and the mechanisms underlying the multiple stress resistance. The combined phenotypic and genotypic approach led to new insights in the mechanisms of increased stress resistance of *L. monocytogenes* LO28 variants. Our findings emphasize the genotypic diversity within the *L. monocytogenes* population and indicate that different types of stress lead to the selection for different types of multiple stress resistant variants.

## Materials and Methods

### Bacterial Strains and Culturing Conditions

*Listeria monocytogenes* LO28 wild type (WT; Wageningen UR Food and Biobased Research, The Netherlands) and acid resistant variants (Metselaar et al., 2013) were used in this study. The stock culture was kept in 15% (v/v) glycerol (Fluka, Buchs) at -80∘C, and before the experiments cells from stock were grown for 2 days at 30∘C on BHI agar (Oxoid, Hampshire). A single colony was used to inoculate 20 ml BHI broth. After over night (ON) growth (18–22 h) at 30∘C (Innova 4335; New Brunswick Scientific, Edison, NJ, USA) with shaking at 160 rpm, 0.5% (v/v) inoculum was added to fresh BHI broth. Cells were grown in BHI at 30∘C until late-exponential growth phase (*OD*_600nm_ = 0.4–0.5 reached after 4–5 h) or stationary phase (18–22 h of growth, *OD*_600nm_ ∼2.0). Independent triplicates were performed for each experiment, unless indicated otherwise. For stress experiments, plates were incubated for 5 days at 30∘C to allow recovery of the cells. Results were expressed as reduction in log_10_ cfu/ml by taking the difference in log_10_ cfu/ml at t_0_ and t_x_. For all other experiments, plates were incubated for 2 days at 30∘C.

### Growth Characteristics

The growth rate at 37 and 7∘C was determined by the twofold dilution (2FD) method as described by Biesta-Peters et al. (2010). Briefly, stationary phase cultures were used for this experiment and diluted in BHI broth to an initial concentration of ∼5⋅10^3^ cfu/ml which was confirmed by plating on BHI agar plates. From this culture, four 2FD in BHI broth were made in duplicate in a 100 well honeycomb plate and the final volume in each well was 200 μl. The plate was incubated in the Bioscreen C (Oy Growth Curves AB Ltd, Helsinki, Finland). The Bioscreen C was set to 37∘C or 7∘C with medium shaking and the OD_600_ was measured every 10 or 30 min, respectively. For the 7∘C measurements, the Bioscreen C was placed in a cold room with an ambient temperature of ∼4∘C. This was done up to 3 weeks, until all wells reached an OD_600_ of at least 0.2 (time to detection TTD). The maximum specific growth rate was determined by taking the reciprocal of the slope between the TTD and the ln(N_0_) for the four 2FD. These experiments were done with biological duplicates (7∘C) and triplicates (37∘C).

### Heat Resistance

Heat inactivation experiments were carried out at 55 ± 0.3∘C in a water bath with shaking at 160 rpm (Julabo SW 23, Julabo Labortechnik, Germany). Four-hundred microliter late-exponential phase culture was transferred into 40 ml preheated BHI broth. After 6 min, 1 ml was taken and decimally diluted in peptone physiological salt (PPS; 0.1% peptone and 0.85% NaCl). A separate Erlenmeyer containing BHI broth at room temperature was used for the *t* = 0 measurement. Appropriate dilutions were spiral plated on BHI agar (Eddy Jet, IUL Instruments).

### Hydrogen Peroxide Resistance

Late-exponential phase cells were exposed to 420 mM H_2_O_2_ for 9 min. This time point was based on the inactivation kinetics of the WT and selected from the data points representing the fast inactivation phase preceding tailing, to achieve a significant reduction in plate counts but with final numbers well above the detection limit. The colony count at *t* = 0 min was determined by adding directly 1 ml of the late-exponential-culture into 9 ml PPS. The appropriate amount of 30% (w/v) H_2_O_2_ was added to the late-exponential phase culture and incubated at 30∘C in a shaking water bath. After 9 min 1 ml was taken, added to 9 ml PPS and appropriate dilutions were made. Diluting and plating were done immediately to avoid continuous H_2_O_2_ inactivation in PPS.

### Benzalkonium Chloride Resistance

Resistance of late-exponential phase cells to benzalkonium chloride (BAC) was determined, similarly, as described for hydrogen peroxide. Prior to the experiment, the count at *t* = 0 was determined by serial dilution of the late-exponential phase culture in PPS. Then, 200 μl of a 5 g/L BAC stock solution was added to 50 ml late-exponential phase culture, resulting in a 20 mg/L final concentration. The culture was incubated at 30∘C in a water bath shaking at 160 rpm and after 5 min, a sample was taken and decimally diluted in PPS. Appropriate dilutions were spiral plated on BHI agar.

### Glutamate Decarboxylase Activity

Glutamate decarboxylase activity was tested for both late-exponential phase cells and stationary phase cells. 100 ml culture was used, of which 50 ml was used for *t* = 0 measurements and 50 ml for measurements after exposure to pH 4.0 for 60 min. This was chosen since no inactivation was observed for this conditions (data not shown) but it is described by Karatzas et al. (2012) that the GAD system is activated under these conditions. Cells were harvested by centrifugation at 7000 × *g* and were resuspended in either BHI adjusted to pH 4.0 by 10 M HCl (acid exposed samples) or BHI (*t* = 0 samples). GABA_i_ concentration was determined on cell lysates of *t* = 0 and acid exposed samples and GABA_e_ concentration was determined in the medium of the acid exposed cells. Cell lysates were prepared as follows: 50 ml culture was harvested by centrifugation at 9000 × *g*. The cell pellet was resuspended in 1 ml BHI, resulting in a cell count of ∼10^11^ cells/ml. Cell suspensions (or media) were boiled for 10 min in an oil bath to lyse all the cells. Subsequently, the cell lysates or media were centrifuged in a tabletop centrifuge for 10 min at 9503 × *g*. Five-hundred microliter of the supernatant was transferred to a clean tube and used for GABA determination. A commercial preparation known as GABase (Sigma–Aldrich, The Netherlands) was used to determine the GABA_i_ and GABA_e_ concentrations. GABA was quantified as described by Tsukatani et al. (2005) with modifications of Karatzas et al. (2010) and O’Byrne et al. (2011). To correct for possible differences in cell density and cell size, the GABA results were corrected for total protein content. Total protein determination was done by using the Pierce BCA Protein Assay Kit (Thermo Scientific, The Netherlands). Total protein was extracted using B-PER^TM^ Bacterial Protein Extraction Reagent (Thermo Scientific, The Netherlands) according to the manufacturer’s instructions.

### Motility Testing

The motility of the strains was tested as described previously (Van Boeijen et al., 2010). Briefly, semisolid medium containing 0.25% (wt/vol) agar (Oxoid), 1% (wt/vol) bacteriological peptone (Oxoid), 0.5% (wt/vol) NaCl (Merck), 0.005% (wt/vol) 2,3,5-triphenyltetrazolium chloride (Sigma–Aldrich, The Netherlands) was inoculated by stabbing a single colony into the medium. After 3 days of incubation at 30∘C, motile strains showed a red cloudy pattern as a result of the reduction of 2,3,5- triphenyltetrazolium chloride to formazan caused by bacterial metabolism. The LO28 WT and immotile HHP variant 17 (Van Boeijen et al., 2010) were used as positive and negative control, respectively. Isolates which showed no or reduced motility at 30∘C were retested at 25∘C to confirm reduced motility, since it is know that motility is temperature dependent (Gründling et al., 2004).

### Biofilm Formation

Biofilm formation was studied in BHI at 30∘C under static conditions. ON cultures were inoculated in fresh BHI broth (0.5% v/v) and a polystyrene 12-well plate was filled with 3 ml of this cell culture in duplicate. The polystyrene plates were incubated at 30∘C for 48 ± 2 h. After incubation, the supernatant was removed and the biofilms were washed three times with phosphate buffered saline (PBS). Biofilms were resuspended in 1 ml PBS by rigorous pipetting, serially diluted in PBS and plated on BHI agar. Plates were incubated at 30∘C and counted after 2 days.

### Virulence Indicators

Lysis of the vacuole and thereby escape from the phagosomes, is critical in *L. monocytogenes* virulence (Rabinovich et al., 2012). Phosphatidylcholine phospholipase C (PC-PLC), or lecithinase, production is necessary for the breakdown of the bacterial plasma membrane after invasion in a human host cell, and is therefore considered a virulence factor in *L. monocytogenes* (Vazquez-Boland et al., 1992). PC-PLC activity was measured as described by Coffey et al. (1996). Briefly, 2 μl ON culture was spotted in triplicate on BHI agar plates, containing 3% NaCl (w/v) and 5% (v/v), e.g., yolk. Plates were incubated for 2 days at 37∘C and PC-PLC activity was determined by evaluating the precipitation zone around the colonies. The second factor which is responsible for vacuole escape is the pore-forming hemolysin, Listeriolysin O (LLO; Rabinovich et al., 2012). LLO activity can be measured by degradation of red blood cells. Hemolysis was tested according to the ISO 11290-1 method. Single colonies were streaked on blood agar plates containing 6% sheep blood. Plates were examined after 24 h of incubation at 37∘C and marked either ++ (clear light zone of hemolysis), + (clear light zone under the colony, but not around) or – (no clear zone) for hemolysis.

### gDNA Extraction

Genomic DNA of *L. monocytogenes* ON cultures was isolated by GenElute Bacterial Genomic DNA Kit (Sigma–Aldrich) using the manufacturers protocol for Gram-positives. Instead of the supplied Elution Solution, 10 mM Tris-HCl pH 8.5 was used for the elution step to avoid residues of EDTA which can interfere with the sequencing reaction. gDNA concentration and purity was measured with an Eppendorf BioPhotometer. gDNA was stored at -20∘C until further use in amplicon sequencing and whole genome sequencing.

### Amplification and Sequencing of *ctsR* and *rpsU*

Primers used for amplification of *ctsR* (Van Boeijen et al., 2008) and *rpsU* are listed in **Table 1**. Primers used for the amplification were designed on the genome of *L. monocytogenes* EGDe by Primer3 and checked to also work for LO28. The amplification was performed with REDTaq DNA Polymerase (Sigma-Aldrich), at an annealing temperature of 55∘C and with an elongation time of 80 s in a MyCycler PCR instrument (Bio-Rad, The Netherlands). For variant 14, for which no *rpsU* PCR product was found, another primer pair was designed (*rpsU_14*, **Table 1**). The PCR products (1.2 kb and 900 bp for *ctsR* and *rpsU,* respectively) were purified by QIAquick PCR Purification Kit (Qiagen, The Netherlands) and sent for sequence analysis (Base Clear B. V., Leiden, The Netherlands). Sequences of the variants were compared to the WT sequence in BioEdit (v7.1.3).

**Table 1:** Primers used for PCR followed by amplicon sequencing and primers used for RT-PCR.

Target	Forward primer (5′→3′)	Reverse primer (5′→3′)	Source
**Primers used for PCR followed by amplicon sequencing**
*ctsR*	GCAGGGATAAACGCTGAAAG	ACTCCGGACATCCAACTC	Van Boeijen et al. (2008)
*rpsU*	CGCGTAGTCCTCCACAATGA	GCCAGAGAAGGCGAGGATAG	This study
*rpsU_14*	ACGATTTCATGTTGACGATGC	CGGTCCATTAGCCTTGTTGT	This study
**Primers used for RT–PCR**
*16S*	GATGCATAGCCGACCTGAGA	TGCTCCGTCAGACTTTCGTC	Van Boeijen et al. (2010)
*tpi*	AACACGGCATGACACCAATC	CACGGATTTGACCACGTACC	Van der Veen et al. (2010)
*ctsR*	GATTAATGGTTGCGGCATTG	CAAAGCAACTAACATCGCTCT	Van Boeijen et al. (2010)
*clpC*	AGTCGATGTTTGGCGATGAG	TGGAGGAGCCCCAACTAAAC	Van Boeijen et al. (2010)
*sigB*	GAAGCAATGGAAATGGGAAA	CATCATCCGTACCACCAACA	This study
*gadD2*	AATACCTTGCCCATGCAGTC	GGCTTGGAAATCTTGGATGA	Karatzas et al. (2010)
*gadT2*	CACGGCTAAAATCGCAAAAT	GGAAGCTTCAACAAAAATGT	Karatzas et al. (2010)
*rpsU*	CGTTTAAAGCGACGAAGAGCA	CGGAGGGAGGGAAAGAGAGA	This study

### Gene Expression of Target Genes

Gene-expression was studied in late-exponential phase. 1 ml cell culture was harvested by centrifugation at 13684 × *g* (Heraeus Biofuge Pico). The cell pellet was resuspended in 1 ml TRI reagent (Applied Biosystems) and flash frozen in liquid nitrogen. Samples were stored at -80∘C until RNA extraction. RNA extraction was performed using the Direct-zol RNA MiniPrep kit (Zymo Research) according to the manufacturer’s instructions, including the DNase treatment. RNA quantity was measured on the Nanodrop and the RNA was checked on degradation by gel electrophoresis. RNA samples were stored at -80∘C. cDNA synthesis was performed by SuperScript III reverse transcriptase (Invitrogen). The real-time PCR was performed using SYBRgreen PCR MasterMix (Applied Biosystems) in a Bio-Rad CFX96 RT-PCR machine. The following steps were performed: initial denaturation (4 min at 95∘C), amplification (40 cycles of 95∘C for 15 s, 59∘C for 1 min) and a melting step (65–95∘C with 0.5∘C steps) to confirm a single product was formed. A standard curve was included to calculate the PCR efficiency for each primer pair. Primers used in this study were taken from literature or designed by Primer3 and are listed in **Table 1**. 16S and *tpi* were used as reference genes and evaluated by BestKeeper (version 1; Pfaﬄ et al., 2004). Relative expression ratios were calculated with the pairwise fixed reallocation randomization test using the relative expression software tool (REST; Pfaﬄ et al., 2002).

### Whole Genome Sequencing and Structural Variation (SV) Analysis

Genomic DNA of LO28 WT and variants 3, 7, 9, 10, 12, 14, 15, 17, 21, and 22 was sent to GATC Biotech (Germany) for library preparation and Illumina paired end sequencing. The WT reads were assembled using the Ray assembler (Boisvert et al., 2010) and annotated with RAST (Aziz et al., 2008). For the SV (SNPs and small insertion and deletions) analysis, low-quality ends of reads (Phred score < = 40) were trimmed and reads shorter than 31 bases were removed with Trim Galore^1^ SV analysis was done with the tool ROVAR^2^, which uses BLAST-like alignment tool (BLAT; Kent, 2002) version 34, to align reads to a repeat-masked reference sequence. A stringent filtering approach was used to minimize false-positive SVs calls: (1) a SNP should be supported by at least one forward read and one reverse read, (2) by at least five unique reads, (3) by at least 20 reads in total, and (4) at least 99% of the reads mapping to the region of the SNP should support the SNP.

### Clustering of Variants Based on Phenotype

**FIGURE 1:**
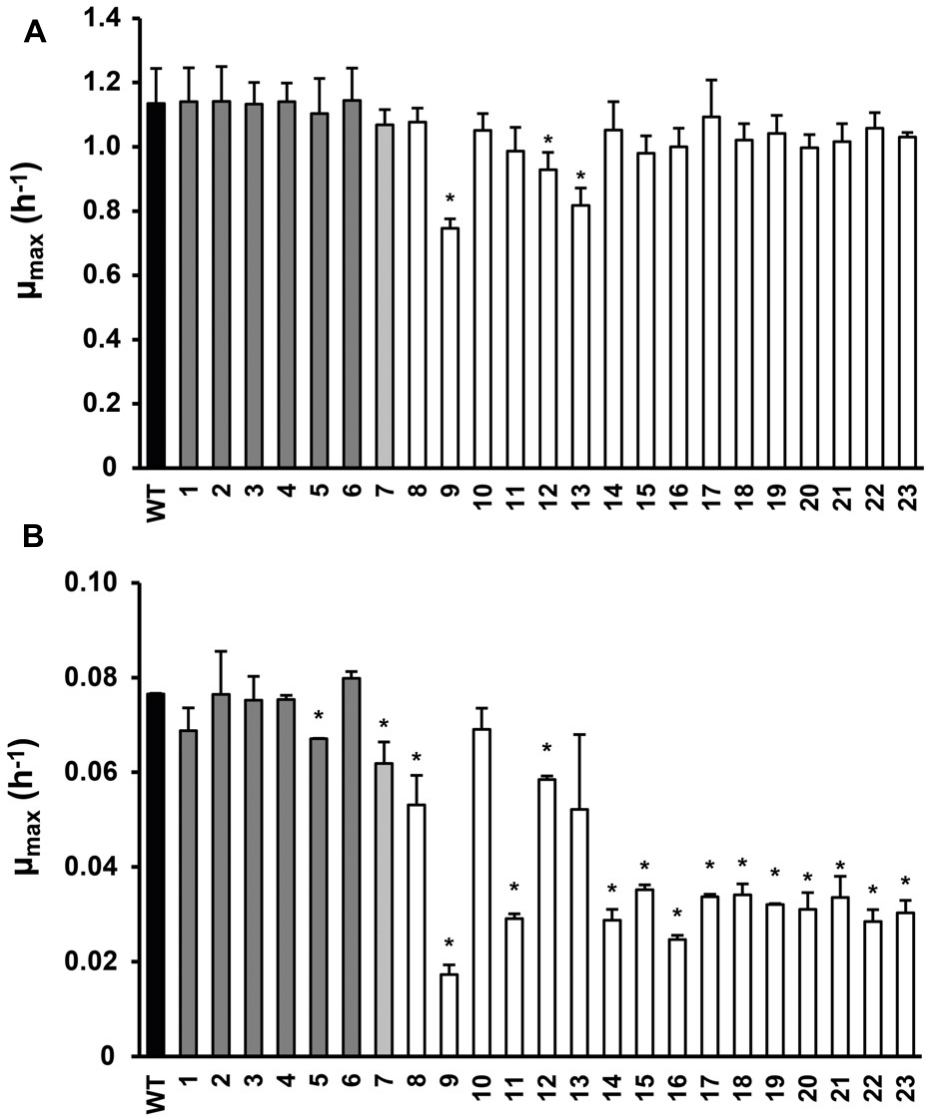
**Maximum specific growth rate of *Listeria monocytogenes* LO28 wild type (WT) and acid resistant variants in Brain Heart Infusion (BHI) at 37∘C (A) and 7∘C (B), determined by the twofold dilution (2FD) method**. Dark gray bars indicate the slightly resistant variants, light gray the variable resistant variant and white the highly acid resistant variants (Metselaar et al., 2013). Error bars represent the SD of the mean for three **(A)** or two **(B)** independent replicates. Significantly different values for μ_max_ compared to the WT are indicated by ∗(*p*< 0.05).

**FIGURE 2:**
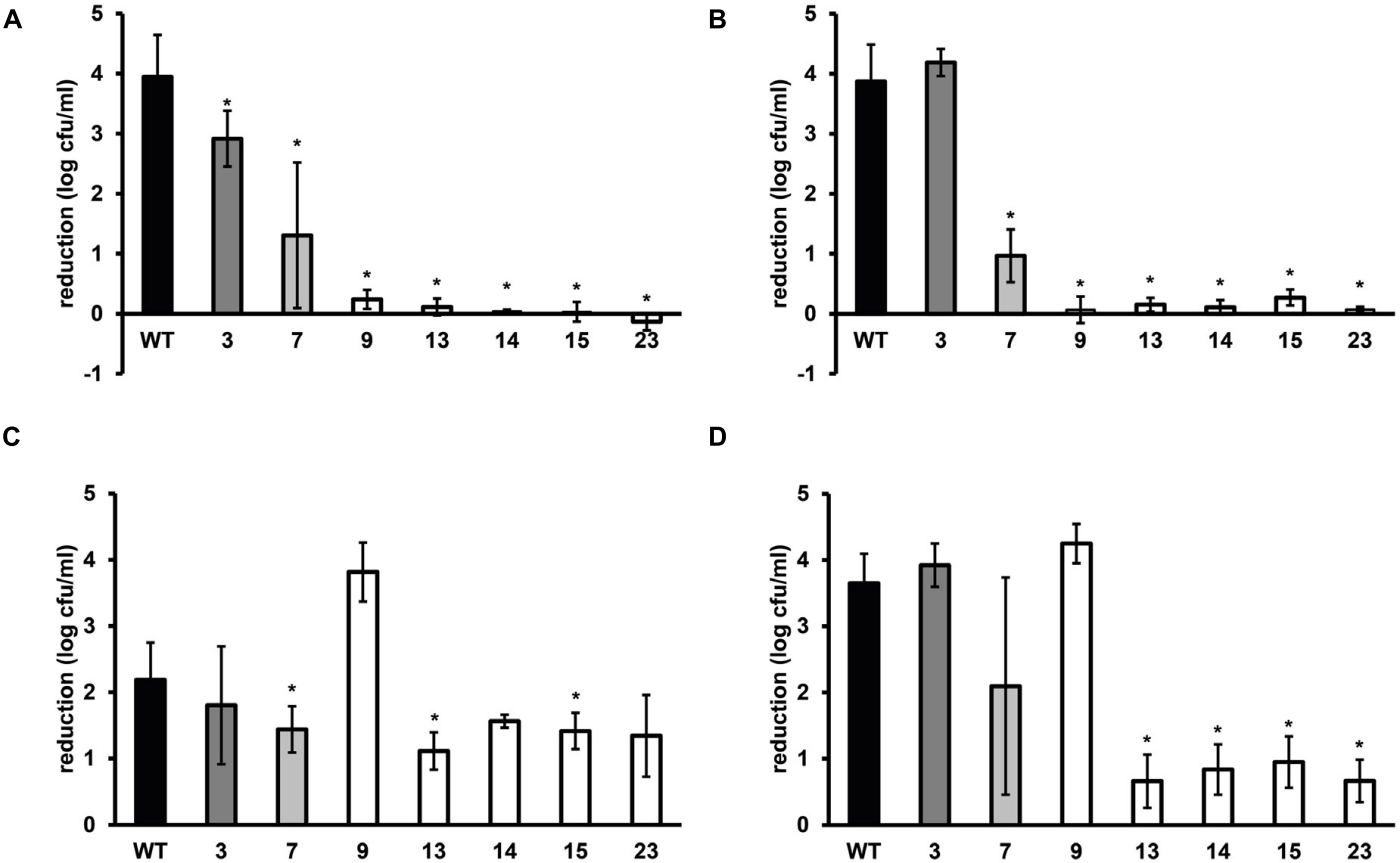
**Stress resistance of late-exponential phase cells of *L. monocytogenes* LO28 WT and acid resistant variants in BHI broth**. Late-exponential phase cells were exposed to pH 2.5 for 3.5 min at 37∘C (adapted from Metselaar et al., 2013) **(A),** 55∘C for 6 min **(B),** 420 mM hydrogen peroxide for 9 min at 30∘C **(C),** and 20 mg/L benzalkonium chloride (BAC) for 5 min at 30∘C **(D)**. Results are expressed as reduction in log_10_ cfu/ml after exposure compared to log_10_ cfu/ml at *t* = 0. Errors bars represent the SD and significant differences from the WT are indicated with ∗(*p* < 0.05).

**FIGURE 3:**
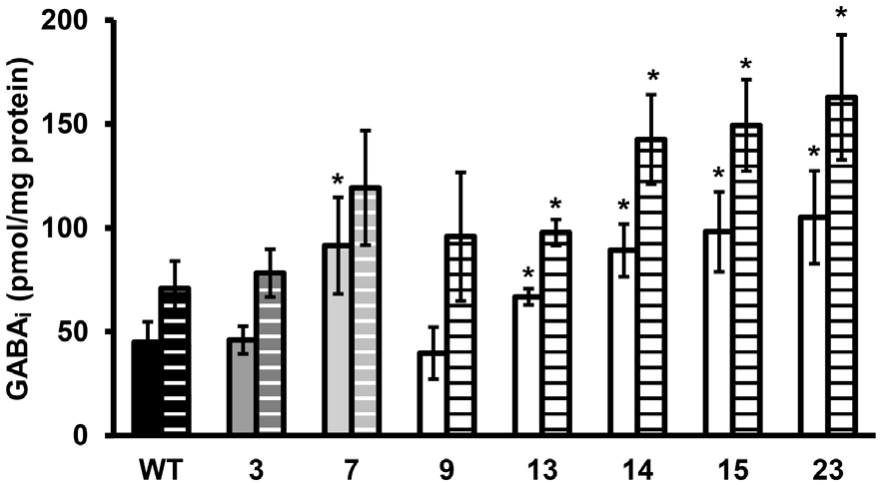
**GABA_i_ concentration of stationary phase cells exposed to pH 4.0 in BHI for 60 min**. Solid bars are the GABA_i_ concentrations at *t* = 0 and dashed bars at *t* = 60 min. GABA_i_ concentrations are corrected for total amount of protein. Error bars include the SD of the GABA and protein measurements and significant differences from the WT are indicated with ∗(*p* < 0.05).

Initial clustering was performed with the WT and a set of seven selected variants (3, 7, 9, 13, 14, 15, and 23) for which a total of 14 phenotypes were determined. The phenotypes that were included in the clustering are: maximum specific growth rate in BHI at 7, 37∘C (**Figure 1**), 30∘C, and in pH 5.0 at 30∘C (Metselaar et al., 2013), acid-, heat-, BAC, and H_2_O_2_ resistance of late-exponential phase cells (**Figure 2**), biofilm formation, motility, hemolysis, and phospholipase activity (**Table 2**), GAD activity (**Figure 3**) and cell size (data not shown). Hierarchical clustering was performed in SPSS (IBM SPSS Statistics 19) by Euclidian distance and within group linkage. Values were standardized on *z*-values. Based on the six phenotypes that were determined for all 23 variants (growth at 7∘C and 37∘C, acid resistance, motility, hemolysis, and phospholipase activity) the remaining 16 variants were added to a matching cluster if possible.

**Table 2:** Motility, biofilm formation, hemolysis, phospholipase-C activity of the variants.

	Motility	Biofilm formation	Phospholipase-C	Hemolysis
LO28 wild type (WT)	++	++	+++	++
Variant 3	++	++	+++	++
Variant 7	++	++	+++	++
Variant 9	++	++	++	++
Variant 13	+	++	+	+
Variant 14	++	++	++	++
Variant 15	++	++	++	++
Variant 23	++	++	++	++

*For motility, biofilm formation, and hemolysis; ++, strongly positive; +, positive. For phospholipase-C: +++, strongly positive; ++, positive; +, positive but weak*.

### Statistical Analysis

A 2-tailed Student’s *t*-test was used to determine statistically significant differences between the WT and variants for the phenotypes. A *p* < 0.05 was considered significant.

## Results

### Growth Characteristics and Selection of Model Variants

Twenty-three stable acid resistant variants of *L. monocytogenes* LO28 were previously divided into three groups, based on their acid resistance: slightly acid resistant, highly acid resistant, and variably acid resistant (Metselaar et al., 2013). The maximum specific growth rate was determined for these 23 variants in BHI broth at 7∘C and at 37∘C (**Figure 1**). At 37∘C variant 9 clearly grew slower than the WT. Also variants 12 and 13 showed a significantly reduced growth rate at 37∘C. The other variants showed similar growth as the WT at 37∘C and the clear trend between acid resistance and growth rate which was established at 30∘C (Metselaar et al., 2013) was not so apparent at 37∘C. However, at 7∘C there was a clear reduction in growth rate for the most highly acid resistant variants. Only variants 10 and 13 of the highly resistant group did not show a significantly lower μ_max_ than the WT, all the other highly resistant variants did. Variants 8 and 12, although displaying a significantly lower μ_max_ than the WT (0.053 and 0.058 versus 0.076 h^-1^, respectively), did not show such a dramatically decreased growth rate as the other variants from the highly resistant group (μ_max_ < 0.035 h^-1^). Variant 7 showed a significantly reduced growth rate. Within the group of slightly resistant variants, only variant 5 had a significantly lower growth rate than the WT. The acid resistance and growth characteristics of the 23 variants were the basis for selecting seven model variants for further detailed phenotypic characterization, namely variants 3, 7, 9, 13, 14, 15, and 23.

### Multiple Stress Resistance and the Role of the Glutamate Decarboxylase System

Late-exponential-phase cells of the selected variants and the WT were exposed to heat, hydrogen peroxide, and BAC. Heat and acid resistance were correlated in these variants (**Figures 2A,B**). The five variants from the highly acid resistant group also showed highly increased survival after 6 min exposure to 55∘C compared to the WT. Whereas the WT shows an almost 4 log_10_ cfu/ml reduction after the exposure time of 6 min, there was less than 0.5 log_10_ cfu/ml reduction for these five variants. Variant 7, showing variable acid resistance, was also significantly more heat resistant than the WT, but with 1 log_10_ reduction less resistant than the highly resistant group. Slightly acid resistant variant 3 did not show significantly increased heat survival. Exposure to the disinfectants hydrogen peroxide and BAC showed a different trend (**Figures 2C,D**) and revealed diversity within the highly resistant group. Variant 9 showed a significantly increased sensitivity to H_2_O_2_ and a similar sensitivity as the WT toward BAC, whereas variants 13, 14, 15, and 23 were significantly more resistant to BAC. Variant 7 showed a variable response toward BAC, as was also observed after acid exposure. The GAD system is one of the most important systems in *L. monocytogenes* to overcome acid stress. Therefore, the activity of the GAD system in the WT and variants was evaluated, by measuring the amount of intracellular and extracellular GABA (GABA_i_ and GABA_e_, respectively), before, and after acid exposure. From **Figure 3** it can be seen that the WT and all variants had a functional GAD system. It was also attempted to measure GABA_e_ levels, but in all cases the concentration was below the detection limit. In all cases the amount of GABA_i_ was increased after an hour exposure to pH 4.0. Variants 13, 14, 15, and 23 displayed higher levels of intracellular GABA, both before and after exposure to pH 4.0 compared to the WT. Variant 7 showed a significantly higher GAD activity before acid exposure but was not significantly different (*p* = 0.052) from the WT after pH 4.0 exposure. Especially when comparing the GABA_i_ levels after acid exposure, it can be seen that variants 14, 15, and 23 showed more than twice the GABA_i_ levels as those measured in the WT. This indicated a possible role of the GAD system in the observed increased acid resistance. However, the increased acid resistance observed in variant 9 was not reflected by an increased GAD activity. On the other hand, the relative increase in GABA_i_ concentration after 1 h exposure to pH 4.0 compared to *t* = 0 was higher for this variant. The expression levels of selected GAD genes *gadD2* and *gadT2* in stationary-phase cells grown in BHI were determined for the WT and variants 3, 7, 9, 13, 14, and 23 by RT-PCR. These genes were chosen as they have been shown to be the major contributors in survival at severe pH stress (Cotter et al., 2005). No significant differences in the expression of these genes between WT and variants was observed.

### Motility, Biofilm Formation, and Virulence Indicators

Motility was tested for all 23 acid resistant variants and all variants were motile at 25 and 30∘C (**Table 2**) suggesting that all the variants possessed flagella, although variants 10 (data not shown), and 13 showed somewhat reduced motility. Some HHP resistant variants lost their motility completely (Van Boeijen et al., 2010), which was apparently not the case for the currently investigated variants. Biofilm formation was tested for the seven selected variants in BHI at 30∘C. Forty-eight hours biofilms were evaluated based on their viable counts (**Table 2**). All variants were capable of forming dense biofilms, with counts higher than 9 log_10_ cfu/well for all variants, and these counts were comparable to the WT. All the variants and the WT were positive for the virulence indicators hemolysis and phospholipase-C. However, both indicators were reduced in variant 13 compared to the WT (**Table 2**).

### Evaluation of the Genetic Basis for Increased Stress Resistance

**FIGURE 4:**
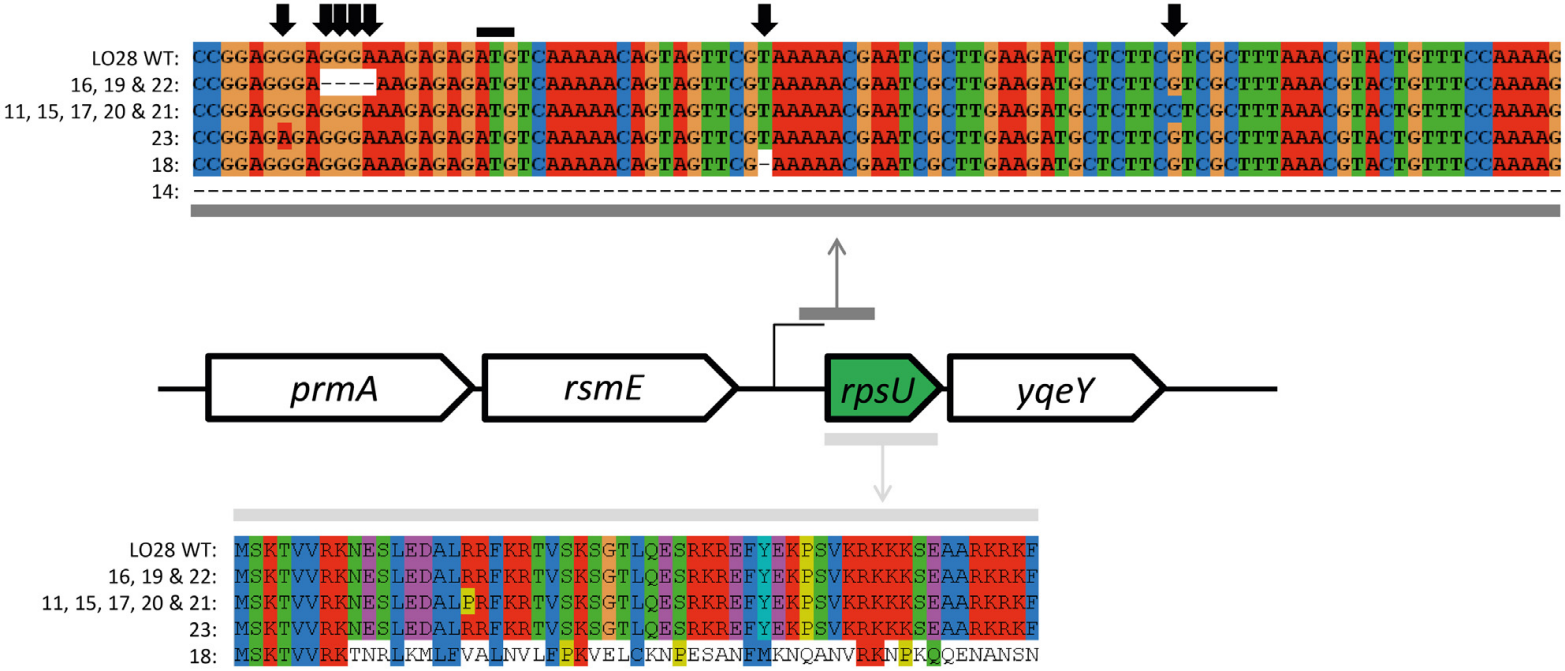
***rpsU* and upstream region sequence alignment of Sanger sequenced *L. monocytogenes* LO28 and acid resistant variants**. The upper alignment represent the nucleotide sequence of the region where mutations were found. The black line indicates the start codon of the *rpsU* gene. The five sequences below the WT are the variations of *rpsU* found in several variants (indicated with the numbers). The lower alignment represents the amino acid sequence of the complete *rpsU* gene and the effect of the mutations on the amino acid sequence.

The *ctsR* and upstream region was sequenced for all the 23 variants. HHP resistant variant 6 (HHP6; Van Boeijen et al., 2010) was included as a control, since this variant is known to have a GGT deletion in the glycine repeat region. The 3 bp deletion was confirmed in this variant. None of the acid resistant variants had a mutation in the sequenced *ctsR* region (data not shown). The sequencing data were confirmed by expression data of *clpC* and *ctsR* of late-exponential-phase cells. None of the seven model variants showed a significant up or down regulation of these genes compared to the WT (data not shown). This is in contrast to the observed upregulation of both *ctsR* and *clpC* of the HHP6 *ctsR* variant (Van Boeijen et al., 2010). Both the sequencing and expression data showed that the increased resistance of the acid resistant variants is not caused by a *ctsR* mutation. Whole genome sequencing of 10 acid resistant variants (3, 7, 9, 10, 12, 14, 15, 17, 21, and 22) and LO28 WT, followed by SV analysis indicated 3 single nucleotide polymorphisms (SNPs) in a total of five variants. Variant 3 and 17 had a T instead of a G at position 2933571. Due to the low quality of the sequence in this region it was not clear whether this SNP was present in an open reading frame (ORF) and therefore not further looked into. Variant 12 had a T instead of a C at position 1411623, which is located in a hypothetical protein. This SNP was confirmed in variant 12 by PCR and amplicon sequencing. This gene of the other variants was also amplified and sequenced, but found to be intact in all 22 other variants. The third SNP was the same mutation (C instead of G at position 1652445) in variant 15, 17, and 21. A nucleotide BLAST search of the ORF where this presumable SNP was present indicated that the annotated gene was *rpsU*, encoding 30S ribosomal protein S21. Amplification of *rpsU* and its promoter region of all 23 variants, followed by sequencing of the resulting PCR products, confirmed an *rpsU* mutation to be present in these three and an additional eight variants. The mutations are visualized in **Figure 4**. Although all 11 variants had a mutation in the same region, five different mutations were identified amongst them. Variants 11, 15, 17, 20, and 21 had the same point mutation, which resulted in an arginine changing into a proline in the protein. Variant 23 had a point mutation in the ribosome binding site (RBS) of *rpsU*. Variants 16, 19, and 22 had a 4 bp deletion, also in the RBS of *rpsU*. Variant 18 had 1 bp deleted in the *rpsU* gene, resulting in a frameshift after eight amino acids. Lastly, variant 14 did not give a PCR product with the *rpsU* primers used (**Table 1**). Another primer pair (*rpsU_14*), aiming at a 1.6 kb product for the WT, resulted in a PCR product of ∼300 bp for variant 14. A 1346 bp deletion, starting 144 bp upstream *rpsU* and covering *rpsU*, *yqeY,* and half of *phoH* was confirmed in this variant after amplicon sequencing. Although different mutations appeared to be present, all these changes in nucleotide sequence affect the RBS or amino acid sequence in such a way that they potentially resulted in a reduction of active S21 protein. Expression of *rpsU* during late-exponential phase was determined for the seven model variants and confirmed that in the *rpsU* variants 14, 15, and 23, expression of *rpsU* was significantly downregulated by 5.8 log_2_ ± 0.001 and 3.9 log_2_ ± 0.010-fold for variant 15 and 23, respectively. Variant 14 did not show any expression for *rpsU* in two out of three replicates and a *C*_t_ > 30 for the third replicate, indicating a severe downregulation in this variant as well. The variants with an intact *rpsU* gene and upstream region showed *rpsU* expression similar to the WT.

### Phenotypic Clustering

**FIGURE 5:**
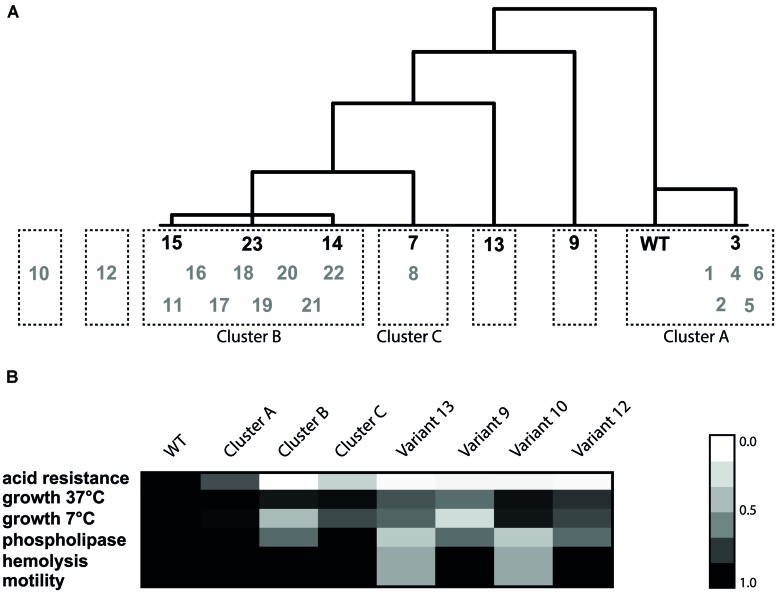
**Hierarchical cluster analysis based on Euclidean distance of the phenotypes **(A)** and heat map of the six phenotypes for each cluster (B)**. The variants in black were the initial selection for cluster analysis and the gray variants were used to validate the resulting clusters. The heat map was made by taking the average of each phenotype within one cluster. Subsequently, the relative ratio of the cluster to the WT was calculated for each phenotype.

Hierarchical clustering was performed on the seven model variants and the WT (**Figure 5**) based on all the 14 measured phenotypic characteristics. The seven variants and WT were divided in five clusters. The first cluster included the WT and slightly resistant variant 3 (Cluster A). This variant only displayed slightly increased acid resistance, but for all the other phenotypes it showed similar behavior to the WT. The second cluster contains variants 14, 15, and 23 (Cluster B). These variants were characterized by increased survival under acid, heat and BAC stress, increased GAD activity, reduced growth rate at 7∘C, normal motility, normal biofilm formation, and normal hemolysis. There were three variants which did not cluster with another variant. Variant 9 was characterized by reduced growth rate under all measured conditions, increased sensitivity to H_2_O_2_ but increased resistance to heat and acid. Variant 7 was very close to cluster B, but with its more intermediate acid resistance, and higher growth rate, not part of this cluster. Variant 13 fell out of the larger cluster, mainly due to its reduced motility, hemolysis, and phospholipase-C activity and the fact that it did not show reduced growth at 7∘C compared to the WT, which was characteristic for cluster B. In order to compare the remaining 16 variants to the seven model variants and to confirm that these seven are representative of the diversity within the population, the phenotypic clustering was extended with the remaining 16 variants. This was done based on the six phenotypic characteristics that were known for all 23 variants (acid resistance, growth characteristics, motility, hemolysis, and phospholipase-C). All slightly acid resistant variants (1, 2, 4, 5, and 6) were grouped in cluster A. The largest cluster (B) consisted of the variants that showed highly increased acid resistance, combined with severely reduced growth rate at 7∘C but unaffected growth rate at 37∘C. Eleven of the 23 variants fell within this cluster B. Interestingly, these are exactly the 11 variants in which a mutation in *rpsU* was identified. Variant 8 did not show significant differences with variant 7 on any of the six phenotypes, despite the fact that variant 7 was initially comprising its own group, due to its variable behavior. These variants formed cluster C. Two variants could not be placed in any of the clusters, namely variants 10 and 12, which mostly show the phenotypes of cluster B, apart from the fact that they do not show the dramatically reduced growth rate at 7∘C which is typical for cluster B. On top of that, variant 12 showed a significantly lower growth rate at 37∘C and variant 10 shows reduced motility, phospholipase, and hemolysis (data not shown). Based on these phenotypes it can be concluded that three clusters and four individual variants were distinguished within the 23 acid resistant variants.

## Discussion

During the transition from soil to human, *L. monocytogenes* encounters several adverse environmental conditions (Freitag et al., 2009). Genotypic diversity within the bacterial population may allow for elevated survival and growth in these different niches. The presence of several small, but highly resistant subpopulations increases the fitness of the population as a whole as it enables survival of several adverse conditions the population might encounter. Our current findings are an important step in unraveling the mechanisms of highly increased resistance of stress resistant variants. The phenotypic characterization indicated a wide diversity within the *L. monocytogenes* LO28 WT population and that different phenotypic characteristics are selected for by applying different types of stress. Based on acid resistance, the 23 variants could be divided in three groups; slightly increased acid resistance, variable acid resistance, and highly increased acid resistance (Metselaar et al., 2013). Although all variants were isolated after acid exposure (pH 3.5 for 90 min), many of the variants were characterized by resistance toward other types of stress as well. The observed phenotypic diversity is in line with the variants isolated after HHP treatment, which also showed a large phenotypic diversity and multiple stress resistance (Van Boeijen et al., 2010). Some of the phenotypic characteristics observed in the HHP resistant variants were observed in the acid resistant variants as well. Heat resistance and acid resistance were strongly correlated in both types of variants, as well as the impaired growth under a range of environmental conditions. However, also differences between HHP- and acid selected variants were observed. Comparison of the phenotypic clusters indicates that the majority of the HHP resistant variants cannot be classified in any of the phenotypic clusters of the acid resistant variants. The possibility of overlapping mechanisms of increased resistance in variants isolated after either HHP stress or acid stress cannot be completely excluded based on the phenotypic characterization alone, but it seems that the majority in both groups of variants is specific to the type of stress after which the variants have been isolated. This was also confirmed by the genotypic characterization. None of the acid resistant variants had a mutation in the *ctsR* gene or upstream region. On the other hand, all 11 members of cluster B were shown to have a mutation in or upstream of their *rpsU* gene, whereas this mutation was not found in the same region of any of the other 12 variants. The clear correlation of this mutation with one phenotypic cluster strongly suggests the mutations in this region to be related to the growth defects and increased stress resistance of this cluster of variants. The fact that these mutations alter the upstream region or affect the amino acid sequence of the gene itself, together with downregulation of *rpsU* in late-exponential phase cells of the *rpsU* variants, suggests a potential role of *rpsU* in stress resistance. The *rpsU* gene encodes the 30S small subunit ribosomal protein S21. In *L. monocytogenes rpsU* is located between *rsmE* (a putative 16S rRNA methyltransferase) and *yqeY* (hypothetical protein). Interestingly, variant 14 had a large deletion, covering *rpsU*, *yqeY,* and part of *phoH* but it clustered phenotypically with the other *rpsU* variants. Apparently, the loss of expression of *yqeY* and *phoH* encoded proteins did not have additional phenotypic effects in the tested conditions. There is not much known about the specific function of ribosomal protein S21 in *L. monocytogenes* or about its role in stress resistance in general, but in *Escherichia coli* and *Bacillus subtilis* some more work has been done. A Δ*rpsU* mutant in *B. subtilis* showed unusual ribosome profiles, a reduced growth rate, and reduced motility (Akanuma et al., 2012). Although motility was unaffected in the *L. monocytogenes rpsU* variants, the reduced growth rate was also observed. The fact that the ribosomal profiles were different in the *B. subtilis* mutants indicated that S21 is an important protein in correct functioning of the ribosomes (Akanuma et al., 2012) but the effect of knocking-out *rpsU* in *B. subtilis* on its stress resistance was not evaluated. Deletion of the genes encoding ribosomal proteins L31 and L25 in *B. subtilis* did result in phenotypes with increased stress resistance (Höper et al., 2005; Reder et al., 2012). Some recent proteome and transcriptome studies in *L. monocytogenes* found differential expression of ribosomal proteins upon stress exposure (Ivy et al., 2012; Durack et al., 2013; Melo et al., 2013; Pleitner et al., 2014). Also, a role in cold adaptation and cold stress response of *L. monocytogenes* has been suggested for specific ribosomal proteins (Bayles et al., 2000; Ivy et al., 2012; Durack et al., 2013). Notably, in other microorganisms expression of S21 was suggested to be temperature-regulated. In the cyanobacterium *Anabaena variabilis*, the transcription of the *rbpA–rpsU* operon was upregulated at low temperature (22∘C) compared to high temperature (38∘C; Sato, 1994) which was correlated with higher levels of protein S21 at low temperature (Sato et al., 1997). In the soil bacterium *Sinorhizobium meliloti rpsU* is located in a cold shock operon with *cspA*. The authors suggest that increased synthesis of S21 might function to compensate for the reduced initiation of translation, caused by the low temperature (O’Connell and Thomashow, 2000). Our data suggest that also in *L. monocytogenes* protein S21 plays a role in growth at low temperature, since all *rpsU* variants show a severely reduced growth rate in BHI at 7∘C compared to the WT. This growth defect is still visible at 30∘C (Metselaar et al., 2013) but restored at 37∘C. The link between *rpsU* and acid resistance or the effect of *rpsU* on the regulation of any of the stress response genes of *L. monocytogenes* has not been studied to our knowledge. One possibility is that the stress resistance of the variants is a direct effect of the reduced growth rate, since it has been reported that reduced growth rate increases the stress resistance of *L. monocytogenes* (Patchett et al., 1996). The inverse correlation between acid resistance of the variants and the growth rate under the conditions at which the cells are grown prior to acid exposure [BHI, 30∘C (Metselaar et al., 2013)] supports this hypothesis. The energy invested in increased stress resistance seems to be at the expense of the growth rate. Whereas in one environment the increased stress resistance might be a major advantage, the reduced growth rate might pose a disadvantage in other competitive environments (Phan and Ferenci, 2013). A reduced growth rate was observed in the *ctsR* mutants (Van Boeijen et al., 2010) as well, most likely as a consequence of the constant activation of the chaperones under the regulation of *ctsR* which is a costly process for the cell. The reduced growth rate can therefore also be considered as a trade-off for the increased stress resistance. One clear phenotype that was observed in not only the three *rpsU* variants but also variants 7 and 13 is the increased GAD activity. The GAD system is one of the most important acid survival mechanisms in *L. monocytogenes.* Although the system, its function and its exact mechanism have been studied intensively over the last years, also many aspects are not clear yet. For *L. monocytogenes* LO28 it is known that the glutamate/GABA antiporters are not active in BHI and that only the intracellular GAD system is responsible for the conversion of glutamate into GABA (Karatzas et al., 2010). In general, increased levels of GABA_i_ have been correlated to increased acid resistance of several *L. monocytogenes* strains. In five of the seven variants in which the GAD_i_ activity was determined, an approximately twofold increase in activity compared to the WT was observed. This is in line with previous data reported by Karatzas et al. (2012) who observed that the 2 log_10_ higher survival upon acid exposure of the WT compared to the *gad* mutants corresponded with around twofold higher GAD activity. Although higher GABA_i_ levels were observed in the variants, this was not reflected by an increased gene expression of the *gad* genes. One possibility is that earlier during growth these genes were transiently higher expressed in the selected acid resistant variants but showing no detectable differences in mRNA levels in stationary phase. Since the *gad* genes are regulated by *sigB* (Wemekamp-Kamphuis et al., 2004), expression levels of *sigB* were determined as well (data not shown) and were not found to be significantly different between WT and variants. Since the GAD activity under the measured conditions solely depends on the GAD_i_ system, and therefore on intracellular pools of glutamate, it can be speculated that the increased GABA concentrations are caused by a higher concentration of intracellular glutamate in these five variants compared to the WT. Until recently, the *L. monocytogenes ctsR* gene appeared to be the main mutation hot spot in natural variants isolated after single stress exposure to heat and high pressure (Van Boeijen et al., 2011). It was surprising that acid exposure did not lead to selection for *ctsR* variants, although according to Van Boeijen et al. (2010), the *ctsR* variants also show increased resistance to pH 2.5 for 3 min. Studying the inactivation kinetics of *ctsR* variant HHP6 at 3.5 showed that the *ctsR* variant is equally sensitive as the LO28 WT (data not shown). Considering the estimated fraction of *ctsR* mutants in the LO28 WT population of 6.5⋅10^-6^ (Van Boeijen et al., 2011), there will be a probability of 1 in ∼100.000 of isolating a *ctsR* mutant after exposure to pH 3.5. This could explain that no *ctsR* variants were isolated after 90 min at pH 3.5 and strengthens the observation that different types of stress, as well as different levels of stress lead to the selection for different types of variants. The large variety of phenotypes that were isolated after randomly chosen durations of stress exposure highlights the large phenotypic diversity within the *L. monocytogenes* population. The combination of phenotypic characterization and whole genome sequencing proved to be a powerful tool in getting new insights in the population diversity of *L. monocytogenes* and possible mechanisms involved in the observed stress tolerance. However, the genetic background for the increased resistance of the other variants remains unclear. This could be due to insufficient read coverage of specific genomic regions or the presence of deletions or insertions larger than about 6 bp that were not taken into account in the SV analysis. To our knowledge, it has not been reported before that a mutation and subsequent down-regulation of one of the ribosomal proteins in *L. monocytogenes* can be advantageous for the bacterial cell under selected conditions by increasing its stress resistance. The trade-off for this increased resistance seems to be the reduced growth ability, especially at low temperature. The impact of these mutations and the corresponding trade-offs on food safety should be studied in more detail. Also, further investigation on a molecular and transcriptome level will provide us with a better understanding of the role of ribosomal proteins in general stress resistance and their role in the generation of genotypic heterogeneity within bacterial populations.

## Conflict of Interest Statement

The authors declare that the research was conducted in the absence of any commercial or financial relationships that could be construed as a potential conflict of interest.

## Footnotes

1 http://www.bioinformatics.babraham.ac.uk/projects/trim_galore/

2 http://trac.nbic.nl/rovar
